# Stem-like and highly invasive prostate cancer cells expressing CD44v8-10 marker originate from CD44-negative cells

**DOI:** 10.18632/oncotarget.25773

**Published:** 2018-07-20

**Authors:** Chiara Di Stefano, Paola Grazioli, Rosaria Anna Fontanella, Paola De Cesaris, Antonella D'Amore, Michele Regno, Donatella Starace, Fabrizio Padula, Micol Elena Fiori, Rita Canipari, Antonella Stoppacciaro, Margherita Pesce, Antonio Filippini, Antonio Francesco Campese, Elio Ziparo, Anna Riccioli

**Affiliations:** ^1^ Department of Anatomy, Histology, Forensic Medicine and Orthopaedics, Section of Histology and Medical Embryology, Sapienza University, Rome, Italy; ^2^ Department of Experimental Medicine, Sapienza University, Rome, Italy; ^3^ Department of Biotechnological and Applied Clinical Sciences, University of L'Aquila, L'Aquila, Italy; ^4^ Department of Oncology and Molecular Medicine, Istituto Superiore di Sanità, Rome, Italy; ^5^ Department of Molecular Medicine, Sapienza University, Rome, Italy; ^6^ Division of Pathology, Sant'Andrea University Hospital, Rome, Italy

**Keywords:** CD44, invasiveness, alternative splicing, prostate stem cells

## Abstract

In human prostate cancer (PCa), the neuroendocrine cells, expressing the prostate cancer stem cell (CSC) marker CD44, may be resistant to androgen ablation and promote tumor recurrence. During the study of heterogeneity of the highly aggressive neuroendocrine PCa cell lines PC3 and DU-145, we isolated and expanded *in vitro* a minor subpopulation of very small cells lacking CD44 (CD44^neg^). Unexpectedly, these sorted CD44^neg^ cells rapidly and spontaneously converted to a stable CD44^high^ phenotype specifically expressing the CD44v8-10 isoform which the sorted CD44^high^ subpopulation failed to express. Surprisingly and potentially interesting, in these cells expression of CD44v8-10 was found to be induced in stem cell medium. CD44 variant isoforms are known to be more expressed in CSC and metastatic cells than CD44 standard isoform. In agreement, functional analysis of the two sorted and cultured subpopulations has shown that the CD44v8-10^pos^ PC3 cells, resulting from the conversion of the CD44^neg^ subpopulation, were more invasive *in vitro* and had a higher clonogenic potential than the sorted CD44^high^ cells, in that they produced mainly holoclones, known to be enriched in stem-like cells. Of interest, the CD44v8-10 is more expressed in human PCa biopsies than in normal gland. The discovery of CD44v8-10^pos^ cells with stem-like and invasive features, derived from a minoritarian CD44^neg^ cell population in PCa, alerts on the high plasticity of stem-like markers and urges for prudency on the approaches to targeting the putative CSC.

## INTRODUCTION

Prostate cancer (PCa) is primarily treated with removal of androgens; however, in the case of primary metastatic PCa, androgen deprivation therapy initially reduces tumor burden, but ultimately the disease will recur in most cases [1]. Literature data on PCa have demonstrated tumour-suppressive functions of TLR3, a receptor for viral double-stranded RNA (dsRNA), and it has been shown that poly(I:C), a synthetic analog of dsRNA, exerts both a direct apoptotic effect on TLR3-expressing cancer cells and anticancer immune stimulation [2, 3]. We have previously demonstrated that poly(I:C) induces apoptosis in the androgen-dependent PCa cell line LNCaP *in vitro* [4] and *in vivo* [5], whereas only a weak apoptotic effect is observed in the more aggressive mCRPC cells PC3 and DU-145 [6]. These cell lines are the prototype of prostatic small cell neuroendocrine carcinoma (SCNC), in which neuroendocrine (NE) features are associated with the expression of the stem/progenitor cell marker CD44 [7]. The hyaluronan receptor CD44 is a single pass transmembrane glycoprotein involved in cell-cell and cell-matrix adhesion. It has a relevant role in lymphocyte homing, inflammation, cell migration and tumour metastasis [8]. CD44 is regarded as a marker of normal prostatic epithelium stem cells as well as cancer stem cells (CSCs) [9] and CD44^high^ PCa cells are more tumorigenic and metastatic than the isogenic CD44-negative (CD44^neg^) PCa cells [10].

To date, there is still no gold-standard to define and identify CSCs in PCa. Traditionally, researchers have isolated prostate CSCs by identifying a combination of cell surface markers, namely CD44 [10], CD133 [11] and α_2_β_1_ integrin [12]. However, PCa is a very heterogeneous tumour in which the CSC pool contains heterogeneous tumorigenic subsets that possess distinct tumour-initiating properties [13].

The present study was initially aimed at testing whether, within the bulk population of very aggressive PCa cell lines, a subset of CSCs could be selected on the basis of different resistance to poly(I:C)-induced apoptosis in analogy with recent data on breast cancer [14]. Unexpectedly, cell separation experiments based on CD44 expression have led us to the identification of a novel cell subpopulation endowed with functional stem like traits. Here we show that in PC3 and DU-145 cell lines this scanty subpopulation includes very small CD44^neg^ cells that rapidly convert to CD44^high^ cells which have high clonogenic and invasive potential and express a specific CD44 variant 3 isoform, characterized by variant exons v8-10 (CD44v8-10), crucial for metastatic feature. Interestingly, CD44v isoforms are expressed in a range of cancers mainly in advanced stages [15] and are associated with stem [16] and metastatic [17] features. In particular, CD44v8-10 is a specific CSC marker of head and neck [18] and gastric cancers [19] and its low expression in normal tissues makes it an ideal target to fight CSCs. Moreover, a close relationship between CD44v8-10 expression and increased metastatic potential has been also demonstrated both in breast [20] and bladder cancers [21]. The high tumorigenic potential of the so far neglected CD44^neg^ subpopulation of PCa cell lines, besides representing an advancement in the dissection of PCa heterogeneity/lineage, strongly highlights the importance of adopting self renewal and metastatic parameters rather than the canonical cell surface markers in the characterization of PCa stem cells.

## RESULTS

### Poly (I:C) treatment selects CD44-negative subpopulation

**Figure 1:**
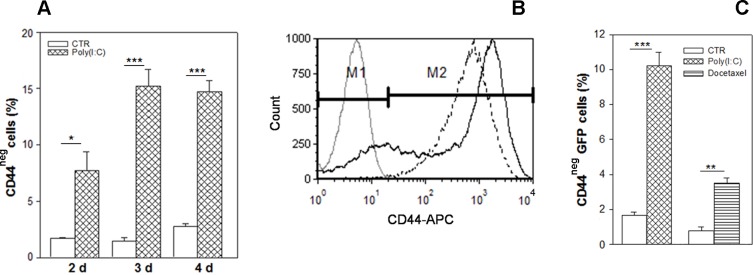
Enrichment in CD44-negative cell subpopulation resulting from Poly(I:C) stimulation **(A)** CD44 expression was evaluated in PC3 cells treated with 25 μg/ml poly (I:C) for 2, 3 and 4 days, calculated as percentage of total live cells. Each data point is the mean ± SEM of three independent experiments ^*^p < 0.05; ^**^p < 0.01, ^***^p < 0.001, Student's paired t-test. **(B)** Representative flow cytometry histogram overlay of live cells before (broken line) and after 72 hours poly (I:C) treatment (continuous line). Poly (I:C) induces an increase of CD44^neg^ cells (M1) and a decrease of CD44-positive population (M2). The grey histogram represents the isotype control APC-labeled IgG2B. Cells were gated using sytox blue stain to exclude dead cells. **(C)** Percentage of CD44-negative cells after 25 μg/ml poly (I:C) or with 20 nM docetaxel in PC3-GFP sorted population (C) and in GFP^low^ cells. Data represent the mean ± SEM derived from three independent experiments ^*^p < 0.05; ^**^p < 0.01; ^***^p < 0.001, Student's paired t-test.

We have previously demonstrated that the androgen-independent cell line PC3 is resistant to poly (I:C)-induced apoptosis [6]. To establish whether such resistance could be correlated to differences in CD44 expression, we treated PC3 cells with 25 μg/ml poly (I:C) for increasing times up to 4 days and analyzed the composition of the cell population by flow cytometry using an anti-CD44 antibody that recognizes both standard and all CD44 variant isoforms. We observed that poly(I:C) treatment resulted in an increased percentage of CD44^neg^ cells (only 2-4% in control sample) up to 5-fold in 3 days treatment (Figure 1A) accompanied by a shift to a median fluorescence intensity (MFI) higher than the control (Figure 1B). The population lacking CD44 protein was very small in size, i.e.~ 60% of these components ranged between 3-7 μm and the others between 7-15 μm (data not shown).

Most of the CD44^neg^-enriched population resulting from poly (I:C) treatment is included in a size area generally comprising the debris in flow cytometry analysis based on forward scatter vs side scatter. Thus, to verify our data excluding debris, we treated with poly (I:C) PC3 cells infected with TWEEN-EGFP lentiviral vector and sorted only the GFP-positive cell population. In addition, to analyze CD44 expression also in cells selected after chemotherapeutic agent, we used docetaxel, which is considered standard first-line therapy in prostate cancer cases following resistance to androgen deprivation therapy. Therefore, we treated the PC3-GFP cells with poly (I:C) or with the docetaxel for 72 hours and flow cytometry analysis confirmed that a high increment of CD44^neg^ population was elicited by both treatments, although less apparent in docetaxel-treated samples (Figure 1C).

### Poly (I:C)-enriched CD44-negative cells give rise to CD44-positive cells

**Figure 2:**
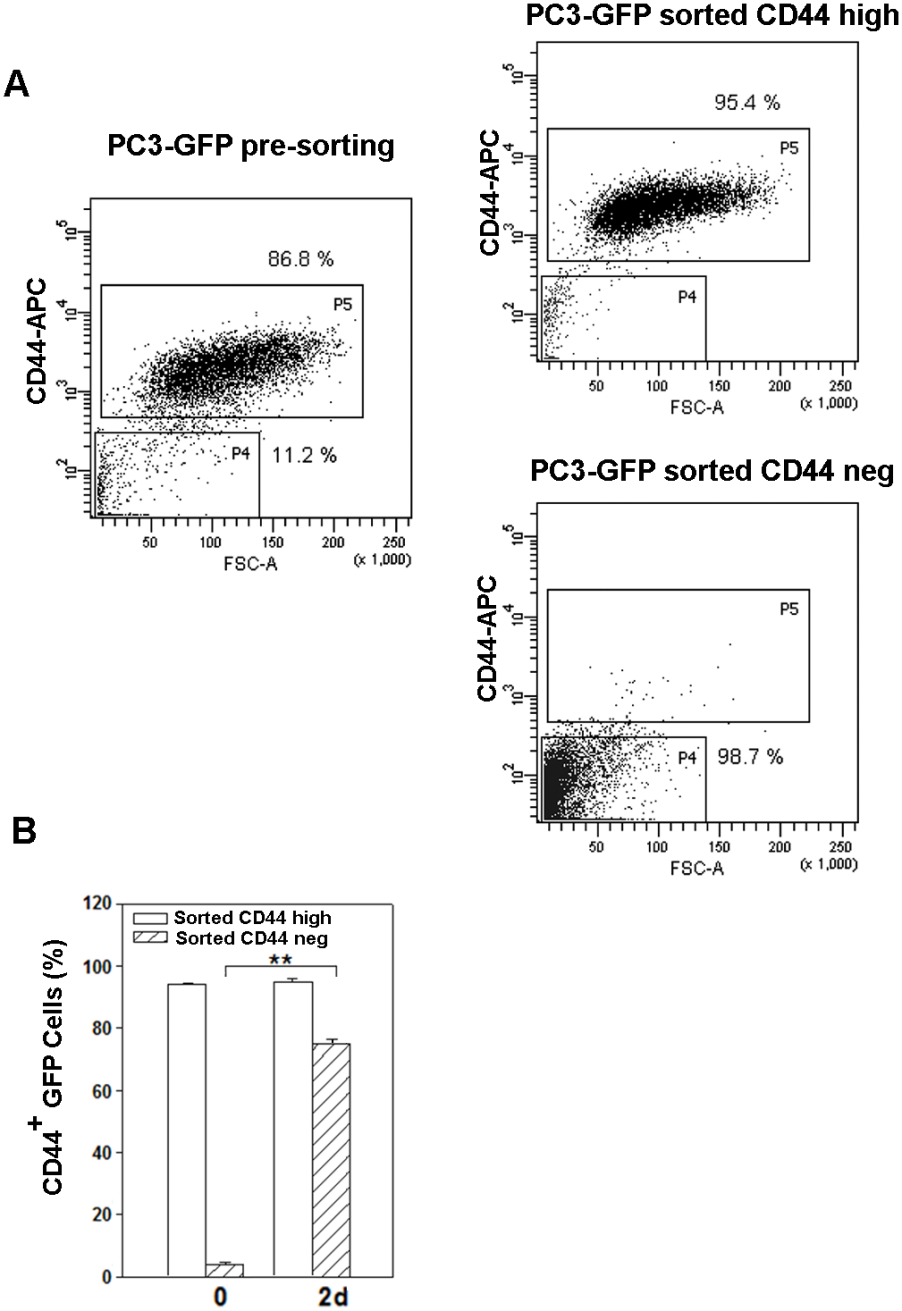
Change in CD44 expression in the two populations sorted from poly (I:C)-treated PC3 cells **(A)** Presorting PC3-GFP CD44^high^ and CD44^neg^ subset distribution after three-day treatment with poly(I:C) (left panel) and CD44^high^ and CD44^neg^ subset purity after sorting from poly(I:C)-treated PC3-GFP (right panels). **(B)** PC3-GFP cells were treated 72 hours with poly (I:C) and sorted for CD44 expression. The histograms represents the percentage of CD44^+^ cells in CD44^neg^GFP and CD44^high^GFP sorted PC3 immediately after sorting (0 d) and two days (2d) after culturing. Cells were gated using sytox blue stain to exclude dead cells. n=3 mean ± S.E.M ^**^p < 0.01, Student's paired t-test.

In order to explore the biological properties of the cell populations resulting from poly (I:C) treatment, single PC3 GFP cell suspensions were obtained and sorted by FACS into CD44^high^ and CD44^neg^ cells. The purity of these cell populations was generally >95% as revealed by post-sort analysis and dot plot data show the small sized CD44^neg^ population (Figure 2A). Surprisingly, 2 days after sorting, the vast majority (75%) of CD44^neg^GFP cells gave rise to a population expressing membrane-CD44 protein (Figure 2B) and showing similar size to sorted CD44^high^ cells (not shown).

### CD44-negative cells sorted from PC3 and DU-145 cell lines spontaneously convert to highly positive CD44 cells

**Figure 3:**
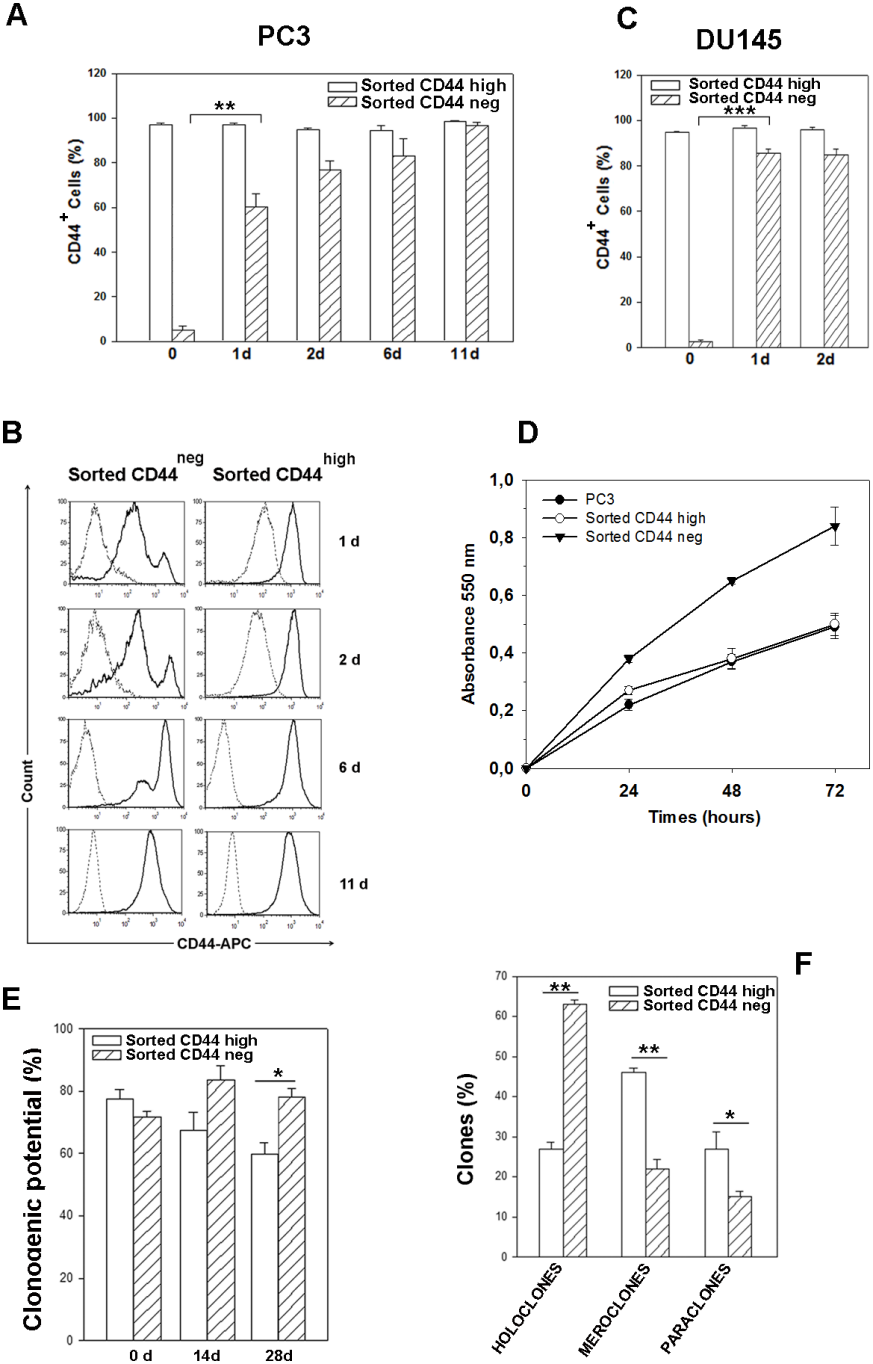
Time course of CD44 expression in CD44neg- and CD44high-sorted cells, proliferation rate and clonogenic potential **(A)** Percentage of CD44^+^ cells in CD44^neg^ and CD44^high^-sorted PC3 populations immediately after sorting (0 d) and for the indicated times after culturing. n=3 mean ± S.E.M ^**^p < 0.01, Student's paired t-test. **(B)** Representative histograms of CD44 expression in CD44^neg^ and CD44^high^ cell subpopulations cultured for the indicated times. The dotted histograms represent the fluorescence of the APC-labeled isotype control IgG2B, the continuous histograms indicate the fluorescence of APC-labeled CD44 antibody. **(C)** Percentage of CD44^+^ cells in CD44^neg^ and CD44^high^-sorted DU-145 populations immediately after sorting (0 d), 1 and 2 days after culturing. Cells were gated using sytox blue stain to exclude dead cells. Results represent the mean from three independent sorting experiments with S.E.M. ^***^p < 0.001, Student's paired t-test. **(D)** Growth curves of cells derived from CD44^neg^ and CD44^high^ cells assayed by MTT. Results represent the mean ± S.E.M of two independent experiments in quadruplicate. **(E)** Assessment of clonogenic potential by limiting dilution assay in CD44^neg^ and CD44^high^ PC3 subpopulations immediately after cell sorting (0 d) and in two expanded populations cultured up to 28 days. Three clonogenic assays performed from three independent sorting experiments were carried out (^*^p < 0.05). **(F)** The clone typing of primary clonogenic assays of the two PC3 sorted populations is shown. Data are from three independent sorting experiments. All the data were analyzed by Student's paired t-test. n=3 mean ± S.E.M.

We next explored whether the minoritarian CD44^neg^ population in parental PC3 cell line could also undergo spontaneous conversion. To this aim, we isolated pure (average, >95%) populations of CD44^high^ and CD44^neg^ cells fractionated by FACS directly from PC3 cultures. Cells from these two PC3 subpopulations were seeded immediately after sorting and monitored by cytometric analysis for CD44 expression over the subsequent 11 days. Purified CD44^high^ cells remained a pure CD44^high^ population, whereas CD44^neg^ cells gave rise to a CD44-positive (CD44^+^) cell population that progressively increased in percentage and in mean fluorescence intensity, in fact, the initial 4% ratio of contaminant CD44^+^ cells in the CD44^neg^ fraction immediately after sorting (0 d) rose to ~ 60% just after 1 day, restoring almost the initial CD44^high^ expression profile in 6 days (Figure 3A and 3B). Of note, neither cell proliferation or cell death occurred in the first 24 hours post sorting, since the number of living cells plated remained constant (not shown), meaning that a fast conversion from CD44^neg^ to CD44^+^ happens and not the selection of a specific cell population.

In addition, similar FACS experiments were performed on DU-145 cells to test whether the fast conversion from CD44^neg^ to CD44^high^ cells was shared by another androgen-insensitive PCa cell line. As shown in Figure 3C, also CD44^neg^ subpopulation sorted from DU-145 spontaneously converted to CD44^high^.

### Cells derived from CD44-negative subpopulation show higher proliferative rate and clonogenic potential than cells derived from CD44^high^ population

Data from literature described CD44-positive cells as stem-like and more tumourigenic cells in prostate cancer [10]. Our results on the conversion of CD44^neg^ sorted cells to CD44^high^ led us to hypothesize that a more aggressive cell population may arise from components of the CD44^neg^ cells. Therefore, in order to investigate and compare the functional properties of CD44^neg^ and CD44^high^ cells, we performed growth curves of cells derived from both cells subpopulations by MTT assay. Data in Figure 3D show that CD44^neg^-derived cells have an enhanced proliferation ability than CD44^high^ and parental PC3 cells. Moreover, we assessed their self renewal potential by evaluating their respective clonogenic potential in limiting dilution assays, as described in Materials and Methods. Although CD44^neg^ and CD44^high^ cells showed a similar clonogenic potential in primary clonogenic assay (Figure 3E, 0 d), after culturing for 28 days, the progeny of CD44^neg^ cells exhibited a significantly higher clonogenic potential than that derived from CD44^high^ cell population which resulted in a decreased clonogenicity (Figure 3E, 28 d). In order to clarify the different clonogenic potential of two cell populations, we analyzed the clone types in primary clonogenic assay, since the formation of holoclones has been adopted as a surrogate stem cell assay, particularly in prostate cancer [22]. In primary clonogenic assay, 2 weeks after plating, 63% ± 1.1 of clones generated by sorted CD44^neg^ cells developed into typical holoclones, 22% ± 1.4 formed meroclones and 15% ± 1.4 formed paraclones. Conversely, the clones derived from sorted CD44^high^ cells were mostly meroclones (46% ± 5.6) and only 27% ± 1.6 were represented by holoclones (Figure 3F). These results explain the decreased clonogenicity of CD44^high^ progeny shown in Figure 3E, because only the holoclones are able to generate the full spectrum of clonal heterogeneity thus ensuring maintenance of the pool of stem-like cells. Furthermore, we verified the holoclone ability to regenerate the full spectrum of clonal heterogeneity selecting 7 holoclones and 3 meroclones derived by CD44^high^ - and CD44^neg^-primary clonogenic assay. In line with previous evidence [23] holoclones, but not meroclones, gave rise to all three types of clones in the secondary clonogenic assay (Supplementary Figure 1).

### The progeny of CD44-negative PC3 subpopulation specifically expresses CD44 v8-10 variant isoform and the epithelial splice factor ESRP-1

**Figure 4:**
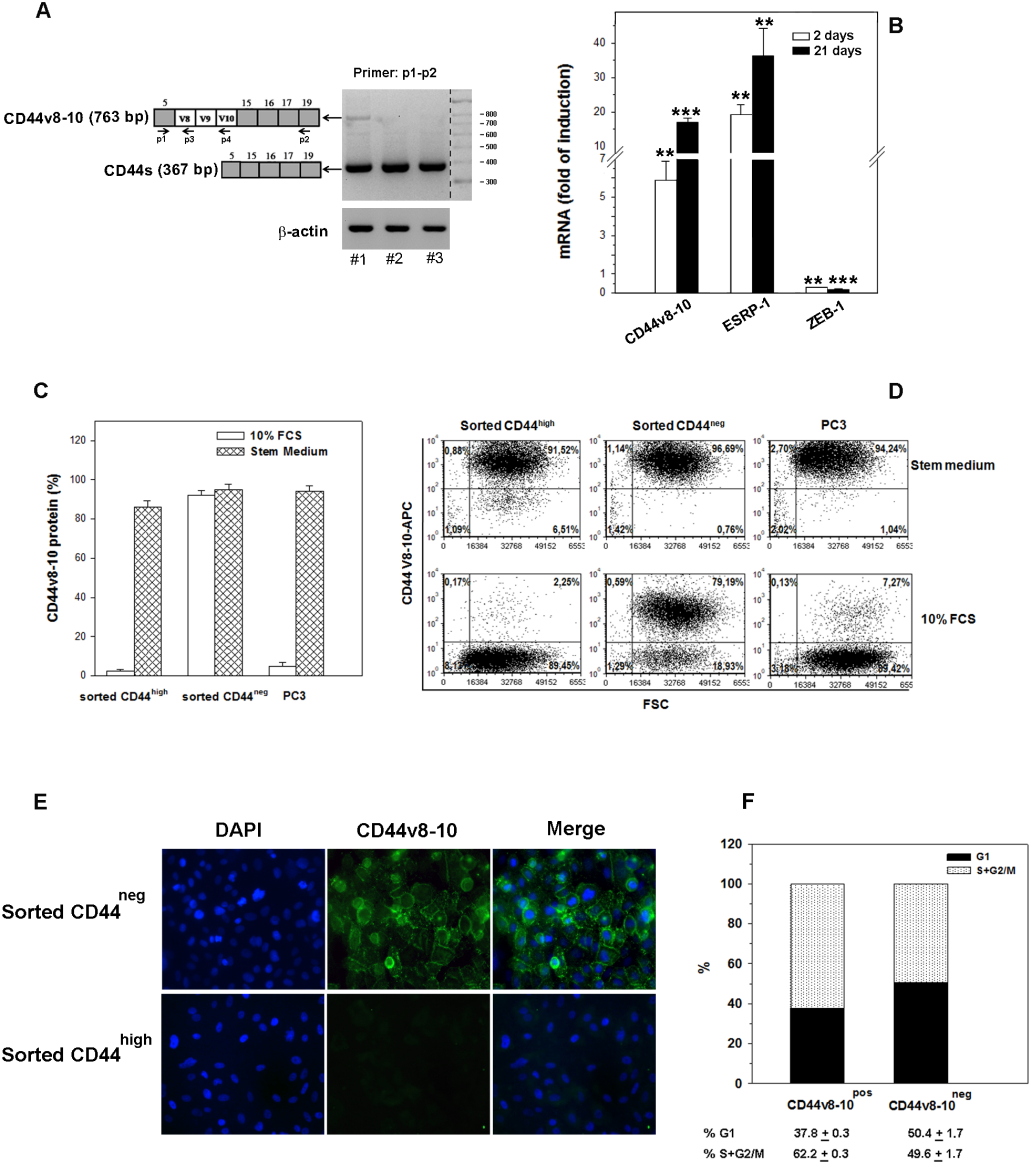
CD44v8-10 expression in CD44neg- and CD44high -derived PC3 populations **(A)** Schematic diagram of human CD44 gene structure showing alternatively spliced variant exons v8-10 in white boxes and constant exons in grey boxes. CD44v8-10 comprises all constant exons and variant exons 8, 9 and 10, while CD44s contains only constant exons. CD44 variant mRNAs were amplified by PCR using the general primers p1 and p2 that can amplify both CD44s (367 bp) and CD44v (763bp). #1, PC3 CD44^neg^ progeny;. #2, PC3 CD44^high^ progeny;. #3 parental PC3. **(B)** qRT-PCR analysis of CD44v8-10, ESRP1 and ZEB1 mRNA in PC3 cells derived from CD44^neg^ and CD44^high^ cell populations 2 and 21 days after sorting. Primers used for qRT-PCR are described in Supplementary Methods. Data are expressed as fold increase in induction of different gene expression calculated by comparing the values of different mRNAs from the progeny of the CD44^neg^ population with those from the progeny of the CD44^high^ population (set arbitrarily at 1). **(C)** Flow cytometry evaluation of the percentage of cells expressing membrane CD44v8-10 isoform in parental PC3 cells and in those derived from CD44^neg^ and CD44^high^ cell populations after culturing in growth medium (10% FCS) or four weeks in stem medium. **(D)** The representative FACS plots show the expression of CD44v8-10 in the same conditions of panel C. **(E)** The representative immunofluorescence analysis of CD44v8-10 protein distribution in cultured PC3 indicated subpopulations. **(F)** Cell cycle analysis of CD44v8-10-positive (CD44^pos^) and CD44-negative (CD44^neg^) PC3 cells was determined. The percentage of cycling cells (S+G2/M) in CD44^pos^ vs CD44^neg^ is 62.2±0.3 vs 49.6±1.7, respectively (p<0.001). Data represent the mean ± S.E.M of three independent experiments.

**Figure 5:**
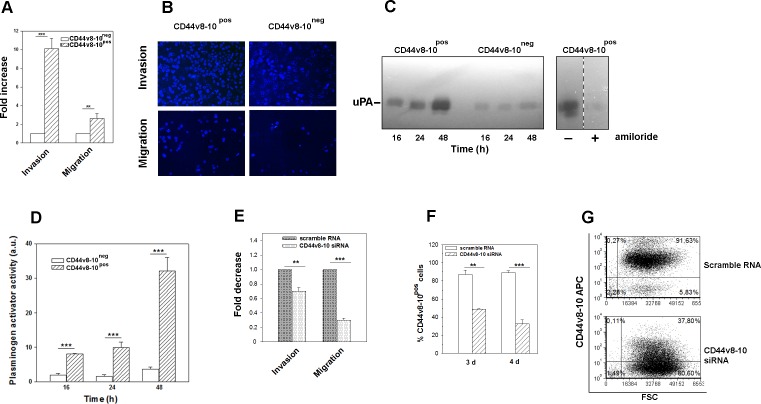
PC3 cells expressing CD44v8-10 display a high invasive potential *in vitro* **(A)** Transwell migration/invasion assays of PC3 subpopulations. Data are expressed as fold increase of migration and invasion relative to CD44v8-10^neg^ cells (set arbitrarily at 1). **(B)** Representative images of migration/invasion rate of DAPI-labeled PC3 different populations. **(C)** Representative zymography of three performed, of PA secreted by CD44v8-10^pos^ and CD44v8-10^neg^ PC3 cells. At the indicated times, conditioned media were collected and analyzed by casein-agar underlay. The lytic zones were plasminogen dependent and were inhibited by 1 mM amiloride, an uPA-specific inhibitor. **(D)** The same conditioned media were assayed for uPA activity by chromogenic substrate assay. Results represent the mean ± SEM of three independently sorted cell subpopulations. (^***^p < 0.001; ^**^p< 0.01). **(E)** Transwell migration/invasion assays of CD44v8-10^pos^ PC3 after specific CD44v8-10 down-regulation by siRNA. Data are expressed as fold decrease of migration and invasion relative to CD44v8-10^pos^ cells treated with scramble RNA (set arbitrarily at 1). Three sample of each subpopulation were examined and the cells counted in seven fields/sample. (^***^p < 0.001). **(F)** Extent of CD44v8-10 knockdown protein after specific CD44v8-10 siRNA or control scramble RNA assayed by flow cytometry. All the data were analyzed by Student's paired t-test. n=3 mean ± S.E.M. **(G)** Representative FACS plots of CD44v8-10 membrane protein expression in CD44v8-10^pos^ PC3 cells four days after specific CD44v8-10 siRNA or control scramble RNA treatments.

The progeny of CD44^neg^ cells after 2 days in culture expressed as much CD44 as the cells derived from the CD44^high^ subpopulation (Figure 3). However, since the CD44 variant isoforms have been linked to subpopulations endowed with stem cell potential, to enhanced metastatic ability and a poor prognosis in several types of cancer [24, 25], we decided to investigate whether the functionally distinct cell populations generated by CD44^neg^ and CD44^high^ cells expressed different CD44 splice variant isoforms at the mRNA and protein level. We first performed semiquantitative RT-PCR from both PC3 CD44^neg^- and CD44^high^-derived cells by designing PCR primers in constant exons 5 and 19 (p1 and p2 in Figure 5A), flanking the variable region of the CD44 gene (Figure 4A). We found a 763-bp product, probably corresponding to CD44 variant 3, containing exons v8, v9 and v10 (CD44 v8-10), exclusively expressed in the progeny of CD44^neg^ cells; while the 367-bp product corresponds to the CD44s mRNA (Figure 4A). In order to evaluate the CD44 v8-10 mRNA levels in the two populations, we performed quantitative RT-PCR (qRT-PCR) analysis with p1 and p3 primers indicated in Figure 4A. Our results show a 20-fold higher expression of CD44 v8-10 mRNA in PC3 CD44^neg^-derived cells compared to CD44^high^-derived population 21 days after sorting (Figure 4B). An enriched expression of CD44 v8-10 transcript was also observed in CD44^neg^-derived DU-145 cells, although at less extent (Supplementary Figure 2A). Since primers p1 and p3 amplify all the CD44 variants that contain the v8 exon, not only CD44v8-10, we verified the sequence of variant exons v8, v9 and v10 by DNA sequencing using primers p1 and p4 (Figure 4A), as described in Supplementary Methods.

We therefore examined the expression of epithelial splicing regulatory protein 1 (ESRP1), which regulates alternative splicing of CD44 mRNA [26]. As expected, we observed that PC3 cells highly expressing CD44v8-10 showed an apparent level of ESRP1 mRNA compared to the progeny of CD44^high^ sorted cells (Figure 4B). Given that ESRP1 is a target of zinc finger E-box binding homeobox 1 (ZEB1) repression, we also found a very low ZEB1 expression in ESRP1-highly positive PC3 subpopulation (Figure 4B).

We next used an antibody specific to CD44v8-10 to assess the differential expression of this variant at the protein level by flow cytometry. About 90% of the PC3 CD44^neg^-derived cells was found to express CD44v8-10 protein, while only 2% of CD44^high^-derived cells expressed this CD44 variant isoform when cultured in 10% FCS medium (Figure 4C). The higher CD44v8-10 protein expression in the progeny of CD44^neg^ compared to CD44^high^ -derived cells was confirmed by indirect immunofluorescence analysis (Figure 4E). Surprisingly and potentially interesting, when the CD44^high^ -derived cells and the parental PC3 cells were maintained in stem medium for four weeks, they displayed high levels of CD44v8-v10 membrane protein (Figure 4C), suggesting that in stem conditions CD44v8-10 expression is strikingly induced. The representative dot plot of the different cell populations is shown in Figure 4D.

Cell cycle analysis by propidium iodide (PI) staining revealed that the proliferation of CD44v8-v10-positive PC3 cells is higher than CD44v8-v10-negative, being the former 62.2% actively cycling (S + G2/M phases) compared to 49.6% of the CD44v8-v10-negative population (Figure 4F).

Moreover, we investigated the amount of CD44v8-10 membrane protein expressed by CD44^neg^-and CD44^high^-derived populations from DU-145 cells. Values of membrane CD44v8-10 protein mirrored that obtained in PC3 cells (Supplementary Figure 2B, 2C).

### CD44 v8-10 expression induces increased motility and invasiveness in PC3 cells

Since we showed that the whole CD44^neg^-derived PC3 population converts to CD44v8-10-expressing population, whereas CD44^high^-derived population does not express this variant, from here onwards we decided to name the first population as CD44v8-10^pos^ PC3, while the latter one as CD44v8-10^neg^ PC3 cells. Therefore, we aimed to investigate the role of CD44v8-10 variant in biological features of CD44v8-10^pos^ PC3 cells. CD44v8-10 was found to define tumour cells with marked metastatic potential [17, 21], thus we characterized migration and invasion capability of the two PC3 subpopulations by using the transwell migration/invasion assays. Our data showed a considerably stronger migration and especially invasion ability of CD44v8-10^pos^ PC3 cells compared to CD44v8-10^neg^ PC3 cells (Figures 5A and 5B). Moreover, activation of proteolytic enzymes necessary to controlled degradation of extracellular matrix is involved in tumour cell invasion. Among these proteases, the Plasminogen activators (PA)/plasminogen system controls proteolysis thus, facilitating tumour invasiveness and growth [27]. Therefore, the presence of PAs in the conditioned media of two subpopulations was evaluated by zymography. Lytic bands appeared in the casein underlay with a mol wt of about 55 kDa, which is the usual size of human urokinase plasminogen activator (uPA) (Figure 5C). The lytic zones were plasminogen dependent. In accordance with their higher invasiveness, CD44v8-10^pos^ PC3 cells secreted higher levels of uPA compared to the CD44v8-10^neg^ population (Figures 5C and 5D).

By using a specific siRNA targeting v9 exon, we demonstrated a key role of CD44v8-10 variant in these invasive features (Figure 5E). CD44v8-10 protein down-regulation after specific siRNA transfection ranged 55-60%, as shown in Figure 5F and 5G.

### CD44v8-10 expression in human PCa samples

**Figure 6:**
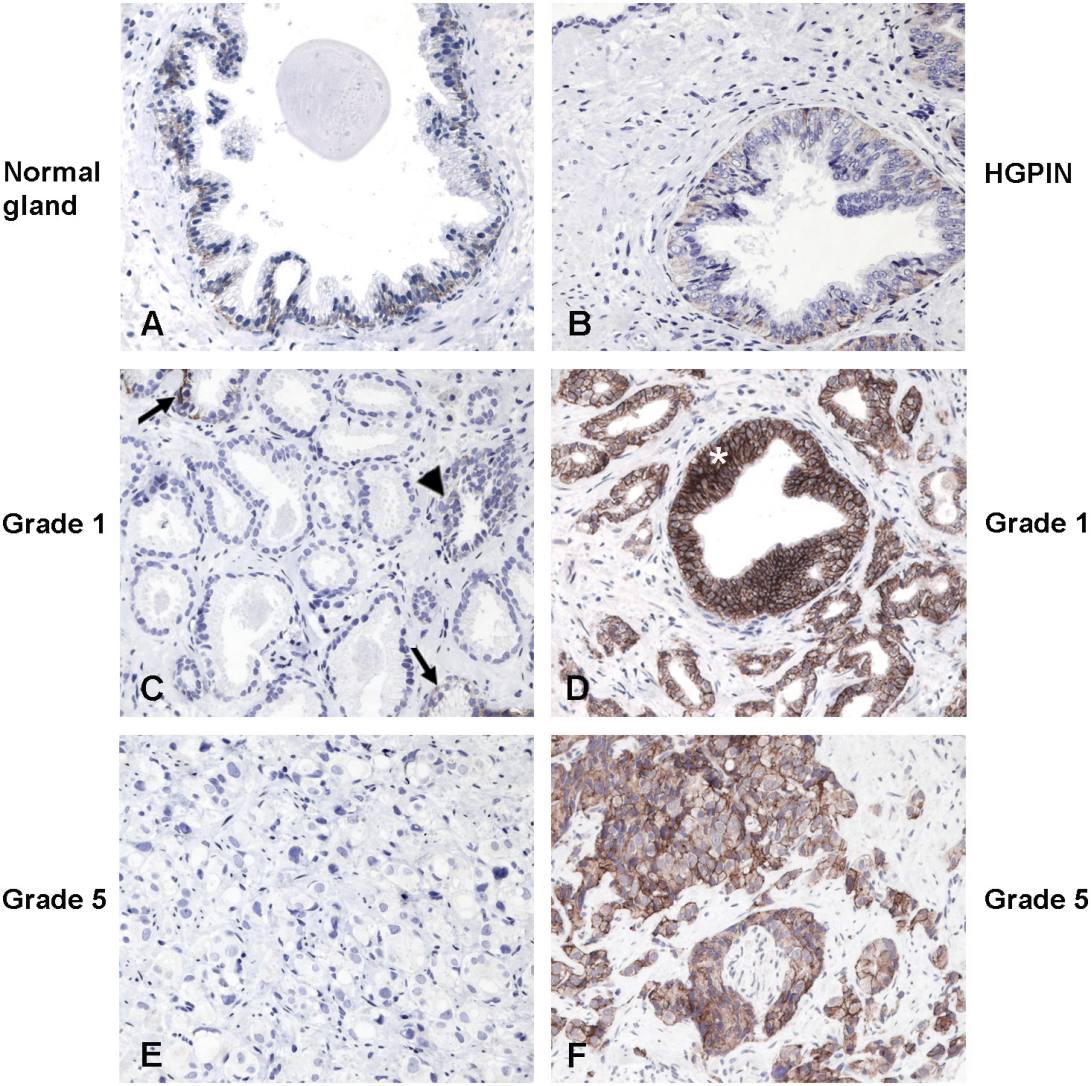
Expression of CD44v8-10 in normal prostate glands and prostate cancers Expression of CD44v8-10 in normal human prostate gland (n=30) and prostate cancer (n=60). **(A)** In normal human glands of non-neoplastic prostate, or entrapped in cancer (C arrows) and in hyperplastic gland (C arrowhead), the expression of CD44v8-10 is limited to the basal cell layer. **(B)** CD44v8-10 expression of HGPIN varies between a weak positivity of the dysplastic cells in the tuft type to the strong membrane staining of the *in situ* tumor of positive tumor cases (D asterisk). Positive **(D, F)** and negative **(C, E)** cases were found in both low group grade (D, E grade 1) or high group grade (F, G grade 5) cancers. Original magnification 200x. Human prostate biopsies were immune-stained as indicated in Materials and Methods.

Although several articles agreed on the fact that a loss of CD44 expression during human prostate cancer progression correlated with higher tumor grade and distant metastasis, recently CD44v6 [28] and v10 [29] have been proposed as useful biomarkers predicting poor outcome following radical prostatectomy for localized PCa. Stated these confusing results on CD44 variants expression in human PCa samples, we analyzed the expression pattern of CD44v8-10 in 30 normal prostate and in prostate cancer tissues from 60 patients using immunohistochemistry. Notably, our analysis reveals that CD44v8-10 reactivity in prostate cancers changes in intensity and distribution when compared to normal prostate tissues. CD44v8-10 expression is limited to epithelial cells but restricted to the membrane of the basal cell layer in normal glands and diffuse to cell membrane of all the displastic cells of the HGPIN, while the expression in positive neoplastic cells is preferentially extended to the cytoplasm and shows a stronger intensity. Residual normal glands in prostate samples behaving tumour show the same distribution of CD44v8-10 as normal prostate. Tumour glands show heterogeneous expression of CD44v8-10 among tumours from the same Gleason group (Figure 6). The small number of cases examined do not establish an evident correlation with Gleason grade or tumour extension (evaluated as n° and percentage of tumour involvement on at least 12 diagnostic biopsies), although showed a trend for increased percentage of CD44v8-10 positive tumours with higher Gleason grade group (Table 1).

**Table 1:** CD44v8-10 expression in primary tumours with different Gleason grades

	Total number	Gleason grade group 1	Gleason grade group 2	Gleason grade group 3	Gleason grade group 4	Gleason grade group 5
N° of primary tumors	60	12	12	12	12	12
N° of CD44v8-10	24	3	3	5	6	5
% positive tumors	40%	25%	25%	42%	50%	42%

## DISCUSSION

The highly heterogeneous nature of human prostate cancer (PCa) both in primary tumour samples and in cell lines poses many obstacles to therapy. Tumour heterogeneity consists of multiple hierarchies with different degrees of differentiation originating from heterogeneous cancer stem cell (CSC) pool [13]. It has been recently demonstrated that stimulation of TLR3 by poly(I:C) promotes breast cancer cells toward a CSC phenotype *in vitro* and *in vivo*, thus potentiating cancer recurrences [14]. Given that CD44 has been proven to be a candidate marker for PCa stem cells [9], the initial aim of the present investigation was to detect whether the apoptosis resistance to poly(I:C) treatment can be linked to CD44 expression and to heterogeneous PCa phenotypes. Unexpectedly, our experimental evidence showed that poly(I:C) treatment induced an enrichment in a CD44^neg^ population besides a CD44 highly positive (CD44^high^) population. Therefore, to explore the tumorigenic features of the two subpopulations selected by poly(I:C), we identified and sorted a CD44^high^ and a CD44^neg^ fractions; with the latter being composed of particularly small cells. We were surprised to find that both CD44^neg^ PC3 cells sorted after poly(I:C) treatment and CD44^neg^ cells from parental PC3 and DU-145 spontaneously converted to CD44^high^ cells. Patrawala and coworkers showed that CD44^high^ PCa cells were more proliferative, clonogenic, tumorigenic, and metastatic than the CD44^neg^ population [10]. On the other hand, they also observed the emergence of CD44-positive highly tumorigenic clones from a pure CD44^neg^ cell population and they explained that tumorigenicity as the ability of a minor subset of cells, within the CD44^neg^ population, to develop into CD44-positive cells. Our results are partially in accordance, however, the selected population defined “negative” by Patralawa represented only the bottom 5% most dimly labelled (CD44^low^), whereas we sorted a subpopulation completely lacking CD44 which represents a highly dynamic and fast-converting subpopulation that expresses CD44 as soon as 24 hours after sorting.

To determine whether the cell populations generated by CD44^neg^ and CD44^high^-sorted cells were functionally distinct, in particular whether CD44^neg^-sorted cells might include “stem-like cells” giving rise to CD44^high^ cells, we performed biological assays and found that CD44^neg^-derived cells displayed a higher proliferative rate as well as a higher clonogenic potential than CD44^high^-derived cells, producing mainly holoclones. Our data suggest that CD44^neg^-derived cells contain a higher proportion of self-renewing cells than CD44^high^-derived cells. Evidence for tumour cell populations that can reversibly shift between stem-like and more committed cells, is emerging. It has been demonstrated that a subpopulation of normal and transformed mammary epithelial CD44^low^ cells spontaneously dedifferentiated into CD44^high^ stem-like cells [30, 31]. Moreover, a cell population lacking CD44 and displaying stem-like traits has also been identified in head and neck squamous carcinoma [32]. Although the conversion from CD44^neg^ into CD44^pos^ cells has been observed in primary mesenchymal stem cells of bone marrow [33], the expression of specific CD44 variants has never been explored. Here we show that CD44v8-10 was the variant isoform predominantly expressed in CD44^neg^-derived cells, whereas it was expressed at lowest levels in the CD44^high^-generated population. CD44v8-10 is described in human colon cancer [34], in rat pancreatic and mammary cancer cells [35] and up-regulated in primary and metastatic tumours but rarely expressed in normal tissue. CD44v8-10 is a gastric CSC marker considered an ideal target to fight these stem cells [19]. Notably, there is evidence that CD44v8-10 enhances the CSC-like characteristics also in prostate cancer. It has been reported that impairing the splicing of CD44v8-10 attenuates the stem-like properties of prostate cancer cells [36].

Moreover, in this paper we show a considerably higher migration and invasion ability of CD44v8-10^pos^ PC3 cells compared to CD44v8-10^neg^ PC3 cells and the down-regulation of CD44v8-10 by siRNA highlights the functional role of this variant in PC3 migration/invasion. In accordance, this variant has been implicated in an enhanced lung metastatic potential in breast cancer cells [20] while CD44v8-10 over-expression into human bladder cancer cells enhances their growth and metastasis *in vivo* [21]. Recently, the poor prognosis of pancreatic adenocarcinoma has been correlated with urokinase Plasminogen Activator (uPA) increased expression and cancer stemness [37]. Accordingly, we observed a notably higher production and activity of uPA by the progeny of CD44v8-10^pos^ cells. Interestingly, a number of studies recently reported that, in addition to epithelial-mesenchymal transition, the mesenchymal-epithelial transition can also induce stem-like properties and increase metastatic potential in cancer cells [38]. We observed a dramatic expression of ESRP1 in CD44v8-10 highly expressing cells. ESRP1 is responsible for the switch from mesenchymal CD44s to epithelial CD44v isoforms [26]. It has been shown that depletion of ESRP1 in metastatic breast cancer cells changed the phenotype of these cells from CD44v to CD44s, and resulted in the suppression of lung metastasis [20].

Moreover, in the late nineties several authors, by using immunohistochemical analysis of human prostate cancer specimens, described a down-regulation/loss of CD44 expression during human prostate cancer progression correlated with higher tumour grade and distant metastasis [39–41]. More recently, the expression levels of CD44s and all its 9 variants analyzed in surgical PCa specimens by qRT-PCR demonstrated that PCa cases are characterized by an over-expression of all the variants. However, during cancer progression, a loss of expression of all CD44 variants was found [42]. Conversely, data showed by Tei et al. suggested that over-expression of CD44v6 could be a useful biomarker predicting poor outcome for localized PCa [28]. In our study, CD44v8-10 immunostaining on primary tumour samples point out a higher CD44v8-10 expression in tumour than in normal glands.

In conclusion, given that many CD44v isoforms are preferentially expressed on cancer cells and involved in tumour progression, these isoforms seem to be better CSC markers than CD44s isoform. The identification of a CD44v8-10 positive population derived from CD44^neg^ PCa cells will open new fields of investigation aimed at finding innovative strategies to selectively target the putative stem-like subpopulation involved in therapy resistance and tumour relapse.

## MATERIALS AND METHODS

### Cell lines and reagents

PC3 (ATTC n. CRL-1435 Lot n. 61777391) and DU-145 (ATTC n. HTB-81 Lot n. 59722255) cells were obtained from the American Type Culture Collection (ATCC, Manassas, VA), authenticated by ATTC in February 2015 and June 2014, respectively and routinely checked for mycoplasma. Cells were cultured as previously described [43]. Transfected PC3 cells with TWEEN-EGFP lentiviral vector were kindly provided by Dr. Haas TL (Istituto Superiore di Sanità, Rome, Italy). Poly (I:C) High Molecular Weight (InvivoGen, San Diego, CA tlrl-pic-5) and docetaxel (Sigma, O1885) were diluted in 3% FCS medium.

### Flow cytometry and cell cycle analysis

Cells were detached and incubated with APC-conjugated CD44 antibody (BD-PharMingen, San Diego, CA, USA 559942) or isotype control APC-conjugated IgG2B (BD-PharMingen) in PBS BSA 1% (Sigma) for 30 minutes on ice prior to flow cytometric analysis. Sytox Blue Stain (Life Technologies, Eugene, OR, USA S34857) was added to exclude dead cells. For CD44v8-10 staining 1×10^5^ cells were incubated with 3 μg/ml anti-human CD44v9 primary antibody (clone: RV3) (Cosmo Bio Co. Ltd, Tokyo, Japan LKG-M001) in PBS 0.2 % BSA for 45 minutes at 4°C. Cells were then incubated with the secondary antibody APC-labeled Goat anti-Rat IgG (H+L) (Invitrogen, Carlsbad, CA, USA A10540) for 30 minutes at 4°C. After washing, cells were assayed using a CyAn ADP flow cytometer (Beckman Coulter, Brea, CA, USA) and data analyzed employing FCS5 express Software (De Novo Software). For cell cycle analysis, after labeling for CD44v9, the cells were fixed with 70% ethanol, a few drops at a time mixing the cells, and incubated on ice for 30 minutes. The cells were centrifuged at 500 x g for 10 minutes, washed once in PBS by centrifugation and resuspended in 1 ml PBS containing 5 μg/ml of propidium iodide. Samples were assayed by CyAn ADP flow cytometer and analyzed employing FCS5 express Software.

### Fluorescence-activated cell sorting (FACS) analysis

For sorting experiments, PC3 and DU-145 cells were stained with APC-conjugated CD44 antibody (BD-PharMingen, San Diego, CA, USA). Cell populations were analyzed post-sorting to ensure purity of sorting before progressing with additional experiments. Apoptotic cells were excluded by elimination of cells positive for Fixable Viability Stain 780 (FSV780) (BD Biosciences, San Jose, CA, USA). FACS was performed with a FACSAria Cell Sorter (BD Biosciences, San Jose, CA, USA). Three sorting experiments for parental PC3 and DU-145 and two experiments for PC3-GFP were performed.

### Immunofluorescence

CD44^high^ and CD44^neg^ sorted PC3 cells grown on 35 mm dishes, fixed 15 min in 4% paraformaldehyde at room temperature (RT) and blocked with 10% BSA for 1 hour. For CD44v8-10 staining, cells were then incubated with 6 μg/ml anti-human CD44v9 primary antibody overnight at 4°C and then incubated 1 hour at RT with 10 μg/ml Goat anti-Rat IgG Alexa Fluor 488 secondary antibody (Thermo Fisher Scientific). Cells were washed in PBS and stained with 600 nM DAPI (Thermo Fisher Scientific). Images were acquired by fluorescence light microscope (ZEISS Axioskop 2 plus).

### Single-cell cloning by limiting dilution

CD44^high^- and CD44^neg^-sorted cells were resuspended in fresh medium to generate a single-cell suspension with a density of 10 cells/ml. Then, 100 μl single-cell suspension was dispensed into each well in a 96-well culture plate. One day after plating, only the wells that contained 1 viable cell were selected, excluding the wells with no cells or with more than one cell. These single-cell wells were maintained in 10% FCS medium and were checked after 14 days to establish clonogenic potential. Then, the resulting colonies were graded on their morphology.

### Migration and invasion assays

To determine the invasive ability of the two PC3 sorted subpopulation, the transwell membrane filters (8-μm pore size) (Falcon) were coated with Matrigel (BD Biosciences). 200,000 cells were seeded in the upper chamber with 1% FCS medium, 20% FCS medium was added to the bottom chamber. Following 48 hours incubation, the cells were removed from the top surface of the membrane. The invasive cells adhering to the bottom surface of the membrane were fixed using 4% paraformaldehyde (Electron Microscopy Sciences) and stained with 600 nM DAPI (Thermo-Fisher Scientific). The total number of DAPI-stained nuclei of invading cells was counted under a fluorescence microscope by using ImageJ software in seven randomly chosen macroscopic fields per membrane.

Cell migration was assayed using a Transwell migration chamber without Matrigel after 24 hours incubation. Each experiment was performed in triplicate and was repeated at least three times.

### Plasminogen activator (PA) assay, gel electrophoresis and zymography

The presence and enzymatic activity of PA were assayed as previously described [44]. Experimental details were shown in Supplementary Methods.

### RNA isolation, RT-PCR, qRT-PCR and siRNA

Total RNA was extracted using TRIzol reagent (Invitrogen) and reverse transcription was performed with High-Capacity RNA-to-cDNA^TM^ Kit (Applied Biosystems-Thermo Fisher, Waltham, MA, USA 4387406). The expression profiling of CD44 isoforms in PC3 and DU cells was analysed by semiquantitative RT-PCR. The expression levels of CD44v8-10, ESRP1 and ZEB1 were determined by Sybr Green qRT-PCR (Bioline, Taunton, MA, USA) performed on cDNA using the StepOnePlus™ Real-Time PCR System (ThermoFisher). Data were analyzed by the ΔΔCt method and GAPDH was used to normalize the expression levels of mRNA. siRNAs were transfected with RNAiMAX (Invitrogen) for 5 h according to the manufacturer's protocol. The siRNA sequences were previously described [36] (synthesized by Bio-Fab Research, Rome Italy). The primers used are reported in Supplementary Methods.

### MTT assay

PC3 cells were cultured into 96-well plates at a concentration of 5×10^3^ cells/well and incubated for 24 h, 48h and 72h and the assay was performed according the manufacturer's instructions.

### Immunohistochemistry for CD44v8-10

The study population consisted of 60 patients with PCa who underwent radical prostatectomy or diagnostic biopsies in 2017, the mean age of the patients was 71+/−12 year.

This study was conducted under approval by the S. Andrea Hospital (n°Prot. n. 228 SA_2016, 12.12.2016 RIF. CE: 4208_2016). Histology specimens were obtained from the histoteque of Sant'Andrea Hospital Pathology Section of Clinical and Molecular Department of ‘Sapienza’ University of Rome, among the PCa blocks. 60 cases from diagnostic biopsies were chosen to have 12 cases for each grade group (from G1 to G5, grade group WHO 2017, see Table 1). 10 of the same cases, two of each group grade, also representative tissue blocks from radical prostatectomy were analyzed. 5 μm paraffin sections deparaffined and antigen retrieved by using a pH9 Dako Retriever solution, were immunostained by a DAKO Autostainer, with EnVisionTM FLEX+ revelation system (Dako ColoradoInc, Fort Collins, Colorado, USA). Anti-human CD44 v9 primary antibody (clone: RV3) was used at the optimal dilution 1:1000. Control for the secondary antibody was done withdrawing the primary antibody. Tissues reactivity was finally compared with the THE HUMAN PROTEINATLAS (http://www.proteinatlas.org/). We chose to conduct the study on recent occurrences of the disease to avoid the possible interference of long paraffin embedding on antigen expression or antibody reactivity. Been less than 1 year old cases the follow-up clinical characterizations were not available yet, we relied on Gleason score, the single most important prognostic factor in prostate cancer, for indication of disease outcome [45].

### Statistical analysis

All numerical data were described as mean ± SEM of at least three independent experiments. Data were analyzed using the two-tailed student t-test. A probability value of 0.05 or less was considered significant.

## SUPPLEMENTARY MATERIALS FIGURES



## Abbreviations

PCa: prostate cancer
CRPC: castration-resistant PCa
TLRs: Toll-Like Receptors
CD44-neg: CD44-negative
PA: plasminogen activator
uPA: urokinase PA
SCNC: small cell neuroendocrine carcinoma
FACS: Fluorescence Activated Cell Sorter
CSCs: cancer stem cells
