# Supplementary material for: Stem-like and highly invasive prostate cancer cells expressing CD44v8-10 marker originate from CD44-negative cells
Source: Oncotarget. 2018 Jul 20;9(56):30905–18. doi: 10.18632/oncotarget.25773 (PMC6089404; doi:10.18632/oncotarget.25773)
Supplement: Supplementary file 1 [file oncotarget-09-30905-s001.pdf]

Stem-like and highly invasive prostate cancer cells expressing CD44v8-10 marker originate from CD44-negative cells

SUPPLEMENTARY MATERIALS

DNA sequencing

PCR products from the progeny of CD44^{neg} PC3 subpopulation were separated by 2% agarose gel using the following CD44 primers: 5'ATCCCAGACGAAGACAGTCC 3' and 5'AAGGTC CTGCTTTCCTTCGT 3', designed in constant exon 5 (indicated as p1 in Figure 5A) and exon v10 (indicated as p4 in Figure 5A), respectively. DNA fragment of our interest (362 bp) was excised by scalpel under UV light and purified by QIAquick Gel Extraction Kit (QIAGEN) according to the maufacturer's instruction. 10 ng of DNA each 100 bp of PCR product was sent to Bio-Fab research for DNA sequencing and was then confirmed using Clustal Omega software alignment tool. Omega software was used for multiple sequence alignment before design specific primers by using Primer 3 software.

Plasminogen activator (PA) assay, gel electrophoresis and zymography

Enzymatic activity of PA was assayed according to the method of Shimada et al., [1] using a chromogenic substrate (substrate d-val-leulys-*p*-nitroanilide) assay. Samples were incubated with plasminogen, and the absorbance generated at 405 nm is related to PA activity, normalized to the value in milligrams of protein present in the culture dish.

To characterize the type of PA present in the sample, aliquots of conditioned medium were separated by 10% sodium dodecyl sulfate polyacrylamide gel electrophoresis (SDS-PAGE) under non-reducing conditions according to the procedure of Laemmli [2]. PA was then visualized by placing the Triton X-100 washed gel on a casein-agar-plasminogen underlay, as previously described [3]. All the bands were plasminogen dependent. Molecular weights were calculated from the position of prestained molecular weight markers (Bio–Rad, Milan, Italy, 161-0373) subjected to electrophoresis in parallel lanes.

RT-PCRand qRT-PCR primers

For semiquantitative RT-PCR the following CD44 primers were used:

5'GCAGCACTTCAGGAGGTTAC3'and 5'GCT CCATTGCCACTGTTGAT3' designed in constant exons 5 and 19. Gene expression levels were normalized to beta actin (5'-CTACAATGAGGTGCGTGTGTGG-3' and 5'-CGGTGAGCATCTTCATGAGG-3').

The primers used for Sybr Green qRT-PCR were as follows:

CD44v8-10: FW 5'-ATCCCAGACGAAGACAG TCC-3' and Rev 5'-ACCTGTGTTTGGATTTGCAGT-3'; ESRP1: FW 5'-CCGACAGTTTAACCAGTCAGT-3' and Rev 5'-TCTTGGAAGCCTCAGGATGC-3' ZEB-1: FW 5'-AAGAAATCCTGGGGCCTGAA-3' Rev 5'-AGGATCATGGTTTTGCTCATTTT-3' GAPDH: 5'-TGCACCACCAACAACTGCTTAG-3' and 5'-GAG GCAGGGATGATGTTC-3'. CD44 variant 3; ESRP1 and ZEB1have been related to their specific RefSeq mRNAs: NM_001001390.1, NM_017697 and NM_030751, respectively. Clustal Omega software alignment tool was used.

REFERENCES

1. Shimada H, Mori T, Takada A, Takada Y, Noda Y, Takai I, Kohda H, Nishimura T. Use of chromogenic substrate S-2251 for determination of plasminogen activator in rat ovaries. Thromb Haemost. 1981; 46:507-10.
2. Laemmli UK. Cleavage of structural proteins during the assembly of the head of bacteriophage T4. Nature. 1970; 227:680-5.
3. Belin D, Godeau F, Vassalli JD. Tumor promoter PMA stimulates the synthesis and secretion of mouse pro-urokinase in MSV-transformed 3T3 cells: this is mediated by an increase in urokinase mRNA content. EMBO J. 1984; 3:1901-6.

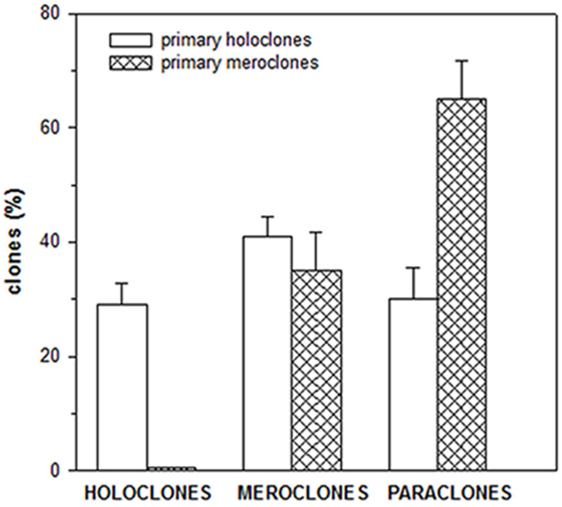

Supplementary Figure 1:Clone classification, following secondary clonogenic assay, of CD44^{neg} and CD44^{high}-derived primary holoclones (n=7) and meroclones (n=3).

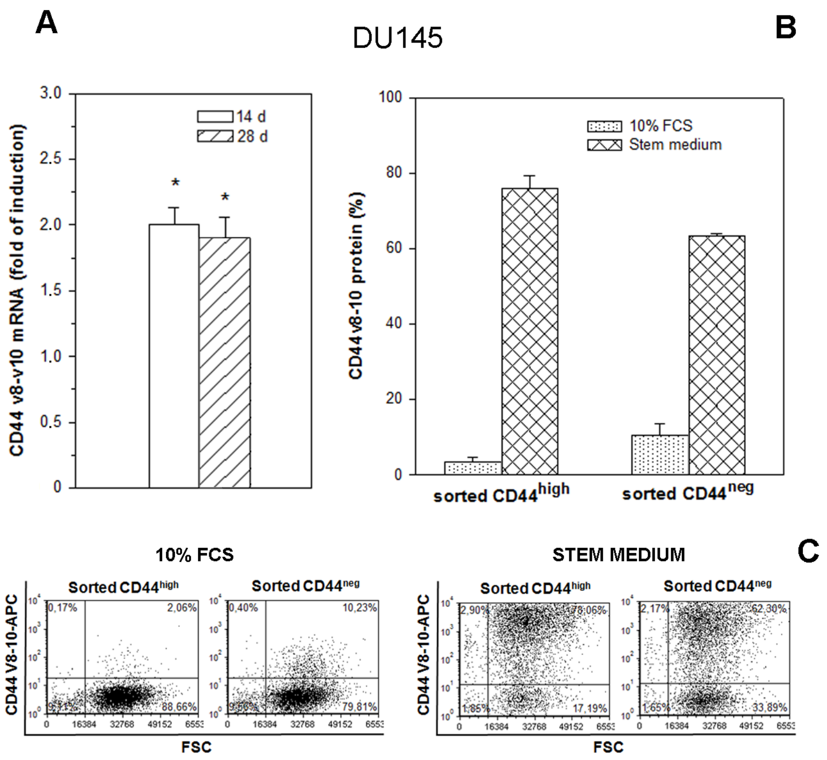

Supplementary Figure 2: DU-145 CD44^{neg}-derived cells express higher levels of CD44 v8-10 transcript **(A)** and membrane protein **(B)** than CD44^{high}-derived cells 14 and 28 days after sorting. Data are shown as fold increase of CD44 v8-10 expression in CD44^{neg}-derived cells compared to CD44^{high}-derived cells (set as 1). All the data were analyzed by Student's paired t-test. n=3 mean ± S.E.M. $^{*}p < 0.05$; $^{**}p < 0.01$. **(C)** The representative FACS plots show a significant higher expression of CD44v8-10 in sorted CD44^{neg} than in sorted CD44^{high} cells.
